# Squamous cell carcinoma-induced gastric conduit-thoracic aorta fistula presenting with massive hematemesis 4 years after radical esophagectomy: a CARE guidelines-compliant case report

**DOI:** 10.3389/fmed.2025.1624035

**Published:** 2025-09-10

**Authors:** Conghua Song, Jianyang Peng, Huiyuan Xu, Xiaomei Li

**Affiliations:** ^1^Gastrointestinal Endoscopy Center, The Affiliated Hospital of Putian University, Putian, China; ^2^Department of Interventional and Vascular Surgery, The Affiliated Hospital of Putian University, Putian, China; ^3^School of Basic Medicine, Putian University, Putian, China; ^4^Key Laboratory of Translational Tumor Medicine in Fujian Province, Putian University, Putian, China

**Keywords:** gastric squamous cell carcinoma, gastric conduit-thoracic aorta fistula, thoracic endovascular aortic repair (TEVAR), digital subtraction angiography (DSA), CARE guidelines

## Abstract

**Introduction:**

Gastric squamous cell carcinoma (GSCC) arising within a gastric conduit is an exceedingly rare phenomenon, and its presentation as a gastro-aortic fistula has never been documented. This case highlights the diagnostic challenges and life-threatening potential of delayed aortoenteric complications after esophagectomy, and underscores the evolving role of endovascular therapy in emergent hemorrhage control.

**Patient concerns and clinical findings:**

A 75-year-old male presented with 6 h of recurrent, high-volume hematemesis and presyncope. On arrival, he was hypotensive (74/50 mmHg), tachycardic, and profoundly anemic (hemoglobin 64 g/L). Physical examination revealed marked conjunctival pallor but a soft, non-tender abdomen without signs of portal hypertension.

**Diagnosis, interventions, and outcomes:**

Emergent computed tomography angiography demonstrated contrast extravasation from the posterior wall of the gastric conduit into the descending thoracic aorta. Digital subtraction angiography confirmed a focal gastro-aortic fistula at the T6 level. Under angiographic guidance, thoracic endovascular aortic repair (TEVAR) was performed using a COOK ZTEG-2PT-30-200 covered stent graft, achieving immediate hemostasis. The patient received massive transfusion support (22 units packed red cells, 8 units cryoprecipitate, 2,000 ml fresh frozen plasma) alongside proton pump inhibitors and somatostatin. Two days post-repair, endoscopic biopsy of the conduit ulcer edge confirmed squamous cell carcinoma. The patient recovered without further bleeding and was discharged day 10 in stable condition. A multidisciplinary tumor board recommended adjuvant chemoradiotherapy. The patient and family opted for palliative care following oncologic consultation due to the advanced disease stage and overall clinical context.

**Conclusion:**

In late post-esophagectomy patients presenting with massive upper gastrointestinal bleeding, high clinical suspicion for arterioenteric fistula is warranted. Computed tomography angiography and DSA should precede endoscopy in hemodynamically unstable patients. TEVAR offers a minimally invasive, rapid means of hemorrhage control, serving as a critical bridge to definitive cancer management.

## Introduction

Gastric squamous cell carcinoma (GSCC) is an exceedingly rare histologic subtype, accounting for only 0.04%−0.5% of all gastric malignancies worldwide (1). GSCC most often affects men in their sixth decade, typically arises in the proximal stomach, and carries a dismal prognosis due to aggressive behavior and late-stage presentation (2). Although gastric tube cancer in post-esophagectomy conduits has been reported-predominantly adenocarcinomas-GSCC arising within a transposed gastric conduit has never been described (3). Aortoenteric fistulae between the gastric conduit and thoracic aorta represent a catastrophic but sporadic late complication of esophagectomy, with historically high mortality rates when managed by open repair (4). In recent years, thoracic endovascular aortic repair (TEVAR) has achieved technical success rates of approximately 87% and 30-day mortality rates below 20%, establishing it as the preferred immediate therapy for life-threatening bleeding (5, 6). Herein, we report the first case of GSCC within a gastric conduit presenting as a gastro-aortic fistula 4 years after esophagectomy, highlighting the need for long-term conduit surveillance and the pivotal role of endovascular management in emergent arterioenteric hemorrhage.

## Patient information

A 75-year-old man presented to our emergency department after 6 hours of recurrent bright-red hematemesis totaling >1,200 ml. He complained of dizziness and weakness but denied chest pain, dysphagia, melena, abdominal pain, or fever. His history was significant for a radical Ivor-Lewis esophagectomy with gastric conduit reconstruction performed 4 years earlier for stage II (pT2N0M0) mid-esophageal squamous cell carcinoma. He did not undergo regular endoscopic surveillance of the gastric conduit due to the stable condition without special discomfort. He had well-controlled hypertension managed with an angiotensin-converting enzyme inhibitor. He had no history of viral hepatitis, portal hypertension, alcohol abuse, or prior peptic ulcer disease. In addition, there was no family history of gastrointestinal malignancies.

## Clinical findings

On initial examination, the patient was alert and oriented but appeared acutely ill and diaphoretic. Vital signs revealed a pulse of 68 beats per minute, respiratory rate of 15 breaths per minute, blood pressure of 74/50 mmHg, and oxygen saturation of 94% on 2 L/min nasal oxygen. He exhibited marked conjunctival and oral mucosal pallor. Cardiopulmonary auscultation was unremarkable, with clear lung fields and regular heart sounds without murmurs. Abdominal inspection showed a flat contour; palpation revealed a soft, non-tender abdomen without rebound or guarding. Bowel sounds were present and normoactive. No peripheral edema or stigmata of chronic liver disease were noted. Continuous nasogastric decompression yielded ongoing dark red fluid, corroborating active upper gastrointestinal bleeding.

## Timeline

The patient, a 75-year-old man with a history of esophagectomy for cancer 4 years prior, presented with acute massive hematemesis and hemodynamic instability. Initial conservative treatment failed, and due to instability, emergency endoscopy was not feasible. CTA and DSA revealed a thoracic aorto-gastric conduit fistula, and emergent TEVAR was performed, successfully stabilizing the patient. Postoperative endoscopy revealed a giant ulcer and biopsy confirmed squamous cell carcinoma at the fistula site. The details are shown in Table 1.

**Table 1:** Clinical timeline of the case.

**Time point**	**Event**	**Key findings/Actions**
Four years before presentation	Transthoracic esophagectomy and gastric conduit reconstruction	Unremarkable recovery; intermittent follow-up
Day 0, 0 h	Onset of massive hematemesis (~1,200 ml) and syncope	Initiation of PPI and somatostatin infusion
Day 0, 2 h	Laboratory: Hb 64 g/L; normal liver and coagulation parameters	Blood product preparation
Day 0, 3 h	Emergent chest CTA	Identification of contrast extravasation at T6 level
Day 0, 5 h	DSA confirmation and TEVAR with COOK ZTEG-2PT-30-200 graft	Immediate hemostasis; massive transfusion (22 U PRBC, 8 U cryoprecipitate, 2000 mL FFP)
Post-TEVAR Day 2	Endoscopic biopsy under sedation	Histopathology confirmed squamous cell carcinoma
Post-TEVAR Days 2–4	Hemodynamic stability; no further hematemesis	Hemoglobin stabilized at ~76 g/L; transfer from ICU
Post-TEVAR Day 10	Discharge to general ward; oncology referral	Planning for definitive cancer management

## Diagnostic assessment

Upon admission, the patient presented with profound hematemesis, hypotension (74/50 mmHg), tachycardia, and signs of hypovolemic shock. Physical examination revealed pallor of the conjunctiva and lips, but no abdominal tenderness or edema. Initial laboratory tests demonstrated severe anemia (Hb 64.0 g/L), with otherwise normal liver function, white blood cell and platelet counts, and negative tumor markers (AFP and CEA), effectively ruling out coagulopathy and portal hypertension-related bleeding. Due to the patient's unstable condition, emergency esophagogastroduodenoscopy (EGD) was not performed.

The clinical suspicion of an aorto-enteric fistula was raised due to recurrent episodes of massive hematemesis and hemodynamic collapse despite conservative management. Emergency computed tomography angiography (CTA) of the chest identified contrast extravasation between the thoracic aorta and the gastric conduit, suggestive of a thoracic aorto-gastric conduit fistula (AGF). This diagnosis was confirmed during digital subtraction angiography (DSA), which demonstrated a direct communication between the aorta and the gastric conduit.

Following successful thoracic endovascular aortic repair (TEVAR), a postoperative EGD revealed a large ulcer at the gastric conduit with visible covered stent material. Biopsy from the ulcer edge showed squamous cell carcinoma (SCC), raising the question of whether the malignancy was primary or secondary in origin, given the rarity of SCC in gastric tissue and the patient's history of esophageal cancer. This added diagnostic complexity, as metachronous recurrence versus primary gastric SCC had to be considered.

Overall, the diagnosis was a thoracic aorto-gastric conduit fistula caused by ulceration from a squamous cell carcinoma at the anastomotic site, presenting with catastrophic upper gastrointestinal bleeding. This case underscores the diagnostic difficulty in recognizing AGF and the value of prompt imaging in critically ill patients. The prognosis in such cases is typically poor without emergent intervention.

## Therapeutic intervention

Upon admission, the patient was immediately started on intensive medical management aimed at stabilizing hemodynamics and controlling upper gastrointestinal bleeding. Pharmacologic interventions included intravenous esomeprazole (80 mg bolus followed by continuous infusion), somatostatin (continuous infusion at 25–50 μg/h), and posterior pituitary hormone (vasopressin analog) to reduce splanchnic blood flow. Concurrently, the patient received aggressive fluid resuscitation and transfusions of packed red blood cells (22 units), cryoprecipitate (8 units), and fresh frozen plasma (2,000 ml) in response to ongoing hemorrhagic shock and severe anemia.

Due to persistent and massive hematemesis, diagnostic angiography was performed. During digital subtraction angiography (DSA), the patient experienced hypotension and altered consciousness, requiring emergency resuscitation. A thoracic endovascular aortic repair (TEVAR) was immediately undertaken using a COOK covered stent graft system (ZTEG-2PT-30-200) to control arterial bleeding. This minimally invasive surgical intervention successfully sealed the aorto-gastric conduit fistula.

Postoperatively, the patient was transferred to the intensive care unit (ICU) for close monitoring. Supportive care included continued intravenous acid suppression, broad-spectrum antibiotics to prevent secondary infection, blood pressure control to maintain a systolic pressure of 80–90 mmHg, and sedation. Mechanical ventilation was provided temporarily via endotracheal intubation. Following hemodynamic stabilization, the patient was successfully extubated, and gastrointestinal decompression showed decreasing bloody output.

No additional surgical procedures were required. The TEVAR procedure proved to be a life-saving intervention that effectively controlled the bleeding source. The multidisciplinary management and rapid implementation of endovascular repair significantly improved the patient's immediate prognosis and provided a window for further diagnostic and oncologic assessment.

## Follow-up and outcomes

Following thoracic endovascular aortic repair (TEVAR), the patient was transferred to the intensive care unit for close monitoring and supportive therapy. Hemodynamic parameters stabilized within 48 h, and gastrointestinal bleeding gradually resolved, with no further episodes of massive hematemesis observed. The patient tolerated the intervention well, with no procedure-related immediate complications.

Two days postoperatively, physical examination revealed improved skin and mucosal perfusion, a normalized level of consciousness, and a stabilized hemoglobin level of 76.0 g/L. Oxygen saturation remained within normal range (96%) with supplemental oxygen. Endotracheal intubation was removed as the patient regained adequate spontaneous respiration, and gastrointestinal decompression output diminished significantly.

**Figure 1:**
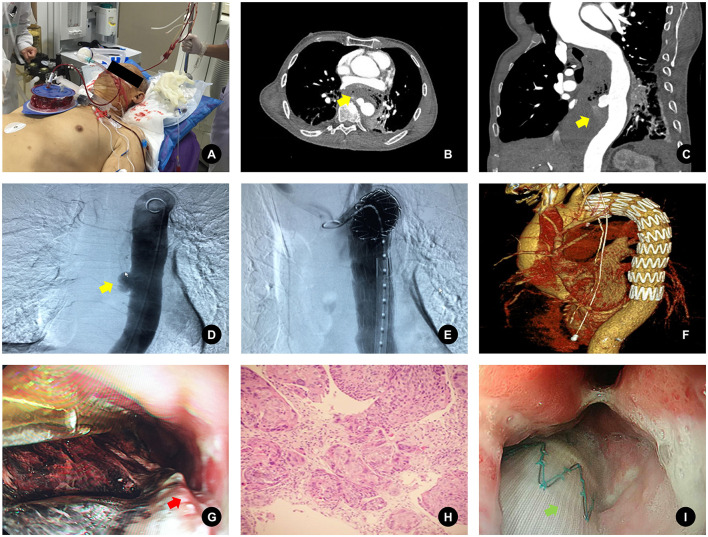
Main diagnostic and therapeutic processes of the case. **(A)** The patient had massive hematemesis on emergency examination. **(B)** Chest axial enhanced CT showed thoracic aortic aneurysm and contrast medium extravasation (yellow arrow). **(C)** Thoracoabdominal aorta CTA shows thoracic aortic aneurysm and contrast medium extravasation (yellow arrow). **(D)** DSA showed thoracic aortic aneurysm-like and contrast extravasation (yellow arrow). **(E)** The DSA guidewire was successfully placed. **(F)** Thoracic endovascular aortic repair (TEVAR) was performed using a COOK ZTEG-2PT-30-200 covered stent graft. **(G)** Gastroscopy on the second day after emergency intervention showed a giant ulcer covered with massive blood clots in the gastric tube and biopsy (red arrow). **(H)** The pathological results of biopsy at the edge of gastric mucosa ulcer under gastroscopy were squamous cell carcinoma. **(I)** Repeat gastroscopy on the 10th day after the intervention showed that the ulcer had been cleaned and the stent was visible inside.

Follow-up esophagogastroduodenoscopy (EGD) revealed a large ulcer within the gastric conduit, located 30–36 cm from the incisors, with visible exposure of the stent graft through the ulcer base. Biopsies obtained from the ulcer margins demonstrated squamous cell carcinoma (SCC) on histopathologic examination. This raised the suspicion of either a rare primary gastric SCC or metastatic recurrence following the prior esophagectomy, given the patient's oncologic history. The details are shown in Figure 1.

The patient was informed of the pathological diagnosis, and multidisciplinary discussions were initiated to determine further oncologic management options. Despite stable immediate post-procedural recovery, the underlying malignancy posed a poor long-term prognosis. No additional adverse events or complications, such as stent migration or infection, were observed during the hospitalization period. The patient and family opted for palliative care following oncologic consultation due to the advanced disease stage and overall clinical context.

## Discussion

Our management was distinguished by the rapid deployment of computed tomography angiography (CTA) to identify the aorto-gastric conduit fistula, followed by immediate thoracic endovascular aortic repair (TEVAR), which achieved prompt hemorrhage control and hemodynamic stabilization. This approach also facilitated early endoscopic biopsy, leading to a definitive diagnosis of squamous cell carcinoma within the gastric conduit. However, the patient's hemodynamic instability precluded emergent endoscopy at presentation, a limitation that underscores the risk of catastrophic bleeding associated with endoscopic evaluation in suspected arterioenteric fistulae. Furthermore, as a single-patient case report, our findings may not be generalizable to all post-esophagectomy bleeding scenarios (3).

Gastric squamous cell carcinoma (GSCC) is an exceedingly rare subtype, comprising only 0.04%−0.5% of gastric malignancies worldwide, with fewer than 100 cases reported to date (1). Metachronous cancer arising in a gastric conduit after esophagectomy is also uncommon, with reported risk factors including mucosal ischemia, bile reflux, *Helicobacter pylori* infection, and prior radiotherapy (7, 8). Aorto-gastric or aortoesophageal fistulae represent catastrophic late complications of esophageal surgery, traditionally managed by high-risk open repair with mortality rates exceeding 50%. These fistulae most commonly result from anastomotic leak, peptic ulceration, or direct tumor invasion (9). In contrast, TEVAR has demonstrated technical success rates of approximately 87.3% and 30-day mortality rates near 19.4%, offering a less invasive and more rapid means of hemorrhage control (10). Comparative analyses indicate significantly lower perioperative mortality with endovascular repair than with open surgery (7.9% vs. 20% at 30 days; OR = 2.94), supporting TEVAR as the preferred initial intervention in unstable patients (11).

The decision to prioritize CTA and DSA over emergent endoscopy was informed by the high risk of exacerbating bleeding with endoscopic manipulation in arterioenteric fistulae, as endoscopic sensitivity for these lesions can be under 25% and may precipitate fatal hemorrhage (12). TEVAR was chosen based on robust evidence of its rapid hemostatic efficacy and lower perioperative risk profile compared to open repair, making it the most appropriate life-saving intervention in this hemodynamically compromised patient (10).

Distinguishing primary GSCC from recurrent esophageal squamous carcinoma is inherently difficult in this patient. The rarity of true GSCC and the prior history of esophageal cancer suggest a possible metachronous recurrence. Histology alone is insufficient, as both share similar morphology. Immunohistochemical markers (p53, CK5/6, p63) confirm squamous phenotype, and molecular comparison with the prior tumor could help assess clonality, but such analyses are rarely available and not definitive. Consequently, the diagnosis remains inferential, yet carries important prognostic and therapeutic implications.

Beyond esophagectomy, arterioenteric fistulae are also recognized as catastrophic complications after vascular surgery, such as aorto-duodenal fistula following abdominal aortic aneurysm grafting. These parallels highlight the importance of maintaining vigilance for arterioenteric bleeding across different postoperative settings. In hemodynamically unstable patients, rapid vascular imaging with CTA should precede endoscopy, and timely endovascular repair remains the cornerstone of life-saving management.

## Conclusion

This case underscores the necessity of maintaining a high index of suspicion for arterioenteric fistula in late post-esophagectomy bleeding, particularly when malignancy is a potential etiology. Rapid vascular imaging should precede endoscopy in unstable patients, and TEVAR should be employed promptly to control hemorrhage. Finally, obtaining histologic confirmation of underlying pathology is essential to guide definitive oncologic management and improve long-term outcomes.

## Patient perspective

Reflecting on my experience, the sudden onset of massive upper gastrointestinal bleeding was both terrifying and bewildering. I had never imagined that such a severe complication could arise years after my esophagectomy. The rapid diagnosis through emergency computed tomography angiography (CTA) and the subsequent thoracic endovascular aortic repair (TEVAR) were life-saving interventions, for which I am profoundly grateful. However, the journey has been fraught with challenges. The physical aftermath, including persistent fatigue and dietary restrictions, has been taxing. Emotionally, grappling with the recurrence of cancer in the form of squamous cell carcinoma in the gastric conduit has been daunting. The uncertainty of prognosis and the need for ongoing surveillance weigh heavily on me. Despite these hurdles, the prompt and decisive medical care I received has been a beacon of hope, underscoring the importance of vigilance and timely intervention in managing complex postoperative complications.

## Informed consent

The patient provided written informed consent for the publication of this case report, including relevant clinical data and imaging findings. The patient understands that all personally identifiable information will be omitted to ensure confidentiality. The patient acknowledges that while complete anonymity cannot be guaranteed, all reasonable efforts will be made to protect their identity. The patient has reviewed the case report and consents to its publication for educational and scientific purposes.

## Data Availability

The original contributions presented in the study are included in the article/supplementary material, further inquiries can be directed to the corresponding authors.
